# Progressive health states and transition probabilities in pediatric dental caries: a systematic review of current evidence and research gaps

**DOI:** 10.3389/froh.2026.1739276

**Published:** 2026-05-01

**Authors:** Xenia Teplitzky, Falk Schwendicke, Laura Neimane, Darja Gostilo, Jekaterina Gudkina, Shaju Jacob Pulikkotil

**Affiliations:** 1 Prof. Shaju Jacob Pulikkotil Group, Oral Health Research, Riga Stradiņš University, Riga, Latvia; 2 Department of Conservative Dentistry and Periodontology, Ludwig-Maximilians-University of Munich, Munich, Germany; 3 Institute of Stomatology, Riga Stradiņš University, Riga, Latvia; 4 Department of General Dentistry, Riga Stradiņš University, Riga, Latvia

**Keywords:** caries progression, health states, markov model, pediatrics, transition probabilities

## Abstract

**Introduction:**

Understanding the progression of dental caries in children, characterized by transitions between distinct health states, may benefit the development of prevention and disease modeling frameworks. This systematic review aimed to synthesize evidence on transition probabilities between childhood dental health states across diverse study designs and populations.

**Methods:**

Comprehensive searches of PubMed and Scopus (last searched September 2025) identified observational and modeling studies reporting discrete caries health states in children. Data were extracted on study design, health state definitions, transition parameters, and model characteristics, and methodological quality was appraised using established tools such as JBI checklists. This review was registered in PROSPERO (CRD420251135660) under the title “Distinct Progressive Health States and Associated Transition Probabilities in Pediatric Dental Caries: A Systematic Review”. The title was modified for clarity in the present manuscript.

**Results:**

A total of 12 reports on 11 studies were included, comprising observational and modeling studies reporting transitions of teeth or surfaces across pediatric populations. Across studies, results were synthesized narratively, most showing that stability within the same health state was the most common pattern, while progression and regression occurred less frequently and were largely dependent on model complexity. Multi-state models capturing lesion depth and activity appear to reflect bidirectional transitions and potential lesion reactivation, whereas simpler two-state models tend to reflect unidirectional onset.

**Discussion:**

Methodological limitations, including bias, variability in lesion assessment, incomplete confounder adjustment, and restrictive modeling assumptions, reduced the robustness and comparability of the evidence. Nevertheless, this review offers supporting data for current model-based research on pediatric caries progression within the scope of this review, highlighting patterns, sources of variability, and the need for harmonized health state definitions, standardized assessment procedures, and transparent reporting to improve future modeling and evidence-based prevention strategies.

**Systematic Review Registration:**

https://www.crd.york.ac.uk/PROSPERO/view/CRD420251135660, PROSPERO CRD420251135660.

## 1 Introduction

Dental caries is a highly prevalent condition in children worldwide and remains a major public health concern despite advances in preventive dentistry. The disease is largely preventable, yet it continues to affect a substantial proportion of the pediatric population and has been described as a “silent epidemic”. According to the 2024 Oral Health Surveillance Report, approximately half of children in the United States aged 6–9 years have experienced tooth decay. Untreated caries in childhood can lead to pain, infection, impaired function, and reduced quality of life. Early caries development carries long-term implications for both oral and general health (1–5).

Dental caries is a dynamic disease characterized by repeated cycles of demineralization and remineralization. Lesions may progress from early enamel changes to cavitated dentin lesions and ultimately to tooth loss if left untreated (6). Because this progression occurs gradually over time and may involve both progression and regression of lesions, understanding the natural history of the disease requires longitudinal approaches that capture changes between clinically meaningful stages.

Modeling disease progression has therefore become an important tool in dental research and health economics. Simulation-based modeling approaches allow researchers to represent the natural history of dental caries using discrete health states (for example, “sound tooth”, “initial lesion”, “cavitated lesion”, or “restored tooth”) and to simulate transitions between these states over time. Such models provide a dynamic, longitudinal view of the disease processes and can be used to evaluate preventive strategies, assess treatment effectiveness, and estimate the long-term costs and health outcomes associated with different intervention scenarios. By combining empirical data with modeling techniques, researchers can project outcomes beyond the duration of clinical studies and explore policy-relevant scenarios that cannot be directly observed in real-world settings.

A key component of disease progression models is the specification of transition probabilities, which quantify the likelihood that an individual or tooth will move from one health state to another within a defined time interval (7). These parameters determine how quickly disease progresses or regresses in a model and strongly influence the estimated impact and cost-effectiveness of preventive and therapeutic interventions. Reported transition probabilities would therefore support producing credible and policy-relevant modeling results.

However, transition probabilities for pediatric dental caries are derived from a wide range of empirical studies that differ in design, populations, follow-up periods, diagnostic criteria, and modeling assumptions. As a result, estimates reported in literature may vary substantially, limiting their comparability and complicating their use as inputs for decision-analytic models. Individual studies often focus on specific populations or contexts and may have limited sample sizes or follow-up durations, which restricts the generalizability of their findings.

A systematic synthesis of the available evidence is therefore needed to identify and compile reported transition probabilities and associated health state structures used to represent pediatric dental caries progression. By systematically reviewing the literature, it is possible to provide a consolidated overview of how health states are defined, how transitions between states are quantified, and where important gaps in the evidence remain. Such synthesis supports the development of more robust disease progression models and improves transparency and comparability across modeling studies.

Although the findings of this review will also inform the development of a future model-based study of childhood caries progression in Latvia, the scope of the present review is global. The aim is to strengthen the evidence base for modeling pediatric dental caries progression and to facilitate the use of consistent and well-documented transition parameters in future research.

This systematic review addresses the following research question: What progressive health states and transition probabilities have been reported in the literature to describe the progression of dental caries in children, and how are these parameters defined and applied across studies?

## 2 Methods

Reporting of this systematic review follows the PRISMA (Preferred Reporting Items for Systematic Reviews and Meta-Analyses) 2020 guidelines (8).

This review was registered in PROSPERO (CRD420251135660) under the title “Distinct Progressive Health States and Associated Transition Probabilities in Pediatric Dental Caries: A Systematic Review”. The title was modified for clarity in the present manuscript. The protocol was included in the registration and can be accessed there.

**Figure 1:**
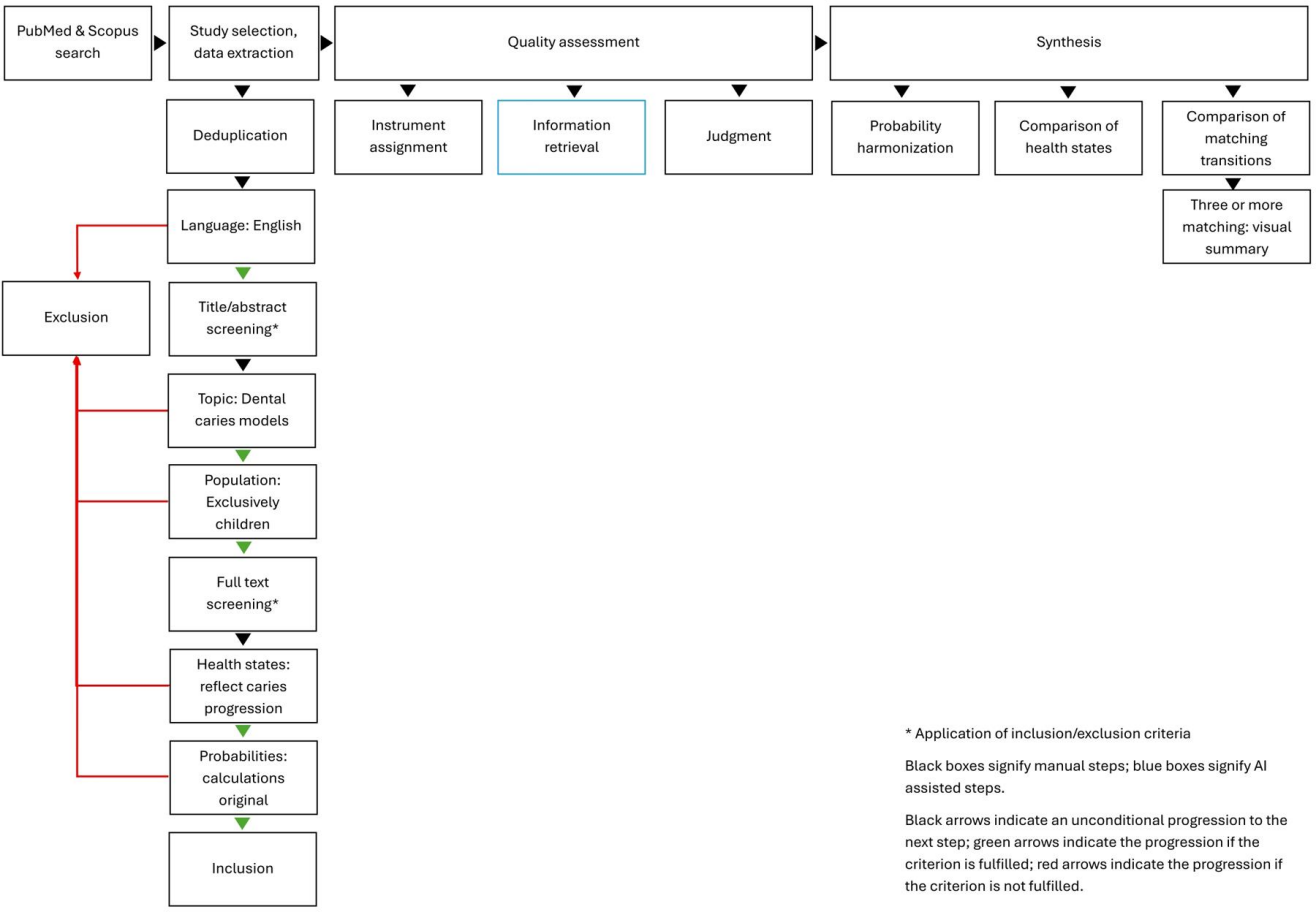
Workflow.

The workflow is documented in Figure 1.

### 2.1 Eligibility criteria

We included studies reporting transitions between dental caries health states in children of any age without comorbidities. Eligible studies provided directly usable information for the calculation of transition probabilities, such as counts, proportions, or percentages of teeth or surfaces moving from one state to another. The PICOS framework—commonly used in systematic reviews to structure research questions and define the eligibility criteria population (P), intervention (I), comparison (C) and outcome (O), and study design (S) (9, 10)—was adapted for this review, as no interventions or comparators were applicable. The population comprised children, and the outcomes were defined as transitions between caries health states.

Both longitudinal studies with baseline and follow-up data and modeling studies using Markov models, transition matrices, or similar approaches were considered. Transition probabilities had to be derived from the study's own data or clearly traceable sources; studies that adopted probabilities from external studies without original calculation were excluded. Health states were defined as actual disease or status categories (e.g., sound, decayed, filled teeth), not interventions or decision-based actions, although studies examining interventions were eligible if these did not form part of the health states. Studies reporting only hazard ratios, regression coefficients, or other indirect measures were excluded. There was no minimum follow-up duration as an eligibility criterion for inclusion. Published studies in English were included, regardless of the publication year.

This approach expands our original protocol in several respects. First, we did not exclude studies based on being available freely or on Elsevier; all but one text identified for full-text review were successfully retrieved, significantly reducing potential publication access bias. Second, we expanded inclusion beyond studies that explicitly defined transitional health states and transition probabilities to include publications providing information directly usable for these calculations. This allowed us to incorporate a broader range of data while maintaining focus on disease progression. Finally, we specified that health state data must reflect caries status only and not treatment decisions; for example, “filled tooth” was considered an acceptable health state, whereas “filling” represented a treatment and was excluded. This criterion preserves comparability even when including studies that examined intervention effects.

For synthesis, studies were grouped according to the terminology and classification of health states used to describe dental caries progression. Because definitions and labeling of health states varied considerably across studies, grouping by terminology allowed for a more coherent comparison of transition patterns within similar conceptual frameworks. This approach facilitated a structured synthesis of the evidence by aligning studies that described comparable stages of caries progression, regardless of study design or modeling approach.

### 2.2 Information sources and search strategy

We searched PubMed (via its native interface) and Scopus (via the Scopus platform) from database inception to 1 September 2025. These two databases are widely used in biomedical and health sciences research. PubMed provides comprehensive coverage of biomedical literature through MEDLINE and related life science journals, while Scopus offers broad multidisciplinary indexing and citation tracking across a wide range of publishers. Together, these databases provide extensive coverage of the relevant literature. ScienceDirect was not searched separately because it primarily functions as a publisher-hosted full-text platform rather than an independent bibliographic database, and most of its journal content—published by Elsevier—is already indexed within Scopus (which is owned by Elsevier). Although broader search engines such as Google Scholar may identify additional records, their search algorithms are not fully transparent, and results may vary between users due to personalization and localization (11). However, reproducibility is a fundamental requirement for systematic reviews (8). Hence, PubMed and Scopus were chosen for this literature search.

The search strategy for PubMed was:

((“Dental Caries”[MeSH Terms] OR “caries”[Title/Abstract] OR “tooth decay”[Title/Abstract] OR “dental decay”[Title/Abstract] OR “dental lesion*”[Title/Abstract]) AND (“Disease Progression”[MeSH Terms] OR “Transition Probabilities”[Title/Abstract] OR “health state transition*”[Title/Abstract] OR “transition state*”[Title/Abstract] OR “markov model*”[Title/Abstract] OR “Markov Chains”[MeSH Terms] OR “state transition*”[Title/Abstract] OR “mutually exclusive health state*”[Title/Abstract] OR “health state model*”[Title/Abstract]) AND (“Child”[MeSH Terms] OR “Pediatrics”[MeSH Terms] OR “Infant”[MeSH Terms] OR “Adolescent”[MeSH Terms] OR “child*”[Title/Abstract] OR “pediatric*”[Title/Abstract] OR “adolescent*”[Title/Abstract] OR “teen*”[Title/Abstract] OR “infant*”[Title/Abstract]) AND (“model*”[Title/Abstract] OR “simulat*”[Title/Abstract] OR “transition probabilit*”[Title/Abstract] OR “state probabilit*”[Title/Abstract] OR “transition matrix”[Title/Abstract] OR “Disease Progression”[Title/Abstract])) AND ((humans[Filter]) AND (english[Filter]) AND (allchild[Filter])).

No further limits were applied.

The search strategy for Scopus was:

TITLE-ABS-KEY (((“dental” W/3 “caries”) OR caries OR (“tooth” W/3 “decay”) OR (“dental” W/3 “decay”) OR “dental lesion*”) AND (“disease progression” OR “transition probabilit*” OR “health state transition*” OR “transition state*” OR “markov model*” OR “markov chain*” OR “state transition*” OR “mutually exclusive health state*” OR “health state model*”) AND (child* OR pediatric* OR adolescent* OR teen* OR infant*) AND (model* OR simulat* OR “transition matrix”)) AND (LIMIT-TO (DOCTYPE, “ar”)) AND (LIMIT-TO (LANGUAGE, “English”))

No further limits were applied.

The search strategies were developed to capture the same conceptual domains across databases but were adapted to the indexing systems and search functionalities of each database. PubMed supports controlled vocabulary through Medical Subject Headings (MeSH), which allows articles to be retrieved using standardized indexing terms in addition to free-text keyword searches. Therefore, the PubMed search strategy combined relevant MeSH terms with free-text keywords restricted to the Title/Abstract fields to ensure comprehensive retrieval of both indexed records and recently published articles that may not yet have been assigned MeSH terms.

In contrast, Scopus does not use MeSH or other controlled biomedical thesauri. Searches must therefore rely exclusively on keyword-based strategies across searchable fields such as title, abstract, and author keywords. Consequently, the Scopus search employed the database-specific field tag TITLE-ABS-KEY and proximity operators (e.g., W/3) to capture relevant variations and relationships between search terms.

As a result of these differences in indexing structure and search syntax, the exact keywords and operators differ between the database-specific strategies. However, both searches were designed to represent the same underlying conceptual components of the research question, namely dental caries, disease progression or state transitions, pediatric populations, and modeling or simulation approaches.

### 2.3 Study selection and data extraction

To determine whether studies met inclusion criteria, titles and abstracts were screened in a first step. Records were either excluded or included for a full text screening (one record's full text could not be retrieved and was excluded). Screening was performed by two independent reviewers (XT, SJP). Any disagreement was solved by consensus. Full details on inclusion and exclusion are contained in Supplementary Tables S1–S6 (Screening). All studies presented sufficient information to decide on inclusion, it was not necessary to obtain or confirm relevant information with study investigators. No translation of articles was required.

Data extraction was also performed by two independent reviewers (XT, SJP) on a pre-agreed and piloted (on five studies) form. Any disagreement was resolved by consensus. All necessary data was available in the records or Supplementary Material; it was not necessary to contact study investigators.

The following items were included:

- Study objective
  ○ Additional to the items specified in the protocol, because the objective may be a major reason for variation across studies.
- Population
  ○ Additional to the items specified in the protocol, because it is plausible that different transition probabilities arise from differences in environment.
- Cycle length
- Duration
- Reasoning for duration (and cycle)
- Determination of health states
- Health states
- Transitions
- Transition probabilities
- Base for probability calculation
- Unit of analysis
- Procedure of assigning the health state to the individual (if applicable)

The primary outcomes of interest were the definitions of health states, the transitions between them, and the corresponding transition probabilities, as these directly inform understanding of disease progression. Additional outcomes were also extracted to support interpretation of the primary outcomes and to explore variability between studies, since differences in context or methodology may influence the estimation of transitions and probabilities.

All transition measures, including counts, percentages, and reported probabilities, were harmonized to a numeric scale ranging from 0 to 1 for consistent interpretation. If several values were provided for a single probability due to factors such as cycle-specific estimates, the minimum and maximum values were reported.

In some cases, transition probabilities were not fully reported. However, missing values could be derived, since the set of possible transitions from a given state must sum to 1 (law of total probability). For example, if the probability of remaining in each state and transitioning to another alternative state were reported, the probability of transitioning to the remaining state could be obtained by subtracting the sum of the reported probabilities from 1.

Because not all included studies contained a model, the items “duration” and “cycle length” (and the reasoning for them) are to be interpreted in a broader sense. While these terms are usually used in the context of state-transition models, we applied them analogously to studies that did not employ a formal model but reported sufficient information to derive changes between health states. For instance, when a study presented baseline and follow-up data indicating the proportion of individuals moving from one state to another, this was treated as representing a transition. In such cases, both duration and cycle length correspond to the time interval between baseline and follow-up, which effectively constitutes a single cycle during which transitions could be observed and quantified.

The full extracted information is contained in Supplementary Tables S15, S17, S19, S21, S23, S25, S27, S29, S31, S33, S35, and S37 (Extraction).

### 2.4 Critical evaluation

Both risk of bias and reporting quality were assessed for all included studies at the study level. Tools were selected according to study design to ensure methodological appropriateness. Risk of bias in modeling studies was evaluated using the ISPOR-SMDM (International Society for Pharmacoeconomics and Outcomes Research—Society for Medical Decision Making) Good Research Practices framework (12) for non-prospective studies and the Quality In Prognosis Studies (QUIPS) tool (13) for prospective studies. Domains IV and V of the ISPOR-SMDM framework were omitted, as they were not applicable to the included models. Cohort and randomized studies were assessed using the Joanna Briggs Institute (JBI) checklists (14, 15). One cohort study with predictive elements was evaluated with the cohort checklist, reflecting the review's focus on observational data rather than predictive performance. The selection of quality assessment instruments was supported by ChatGPT (version GPT-4, model gpt-4-1106, accessed via OpenAI's platform^1^).

Reporting quality was assessed using the Consolidated Health Economic Evaluation Reporting Standards (CHEERS) checklist (16) for non-prospective modeling studies, the Consolidated Standards of Reporting Trials (CONSORT) checklist (17) for the RCT, and the Strengthening the Reporting of Observational Studies in Epidemiology (STROBE) checklist (18) for cohort studies. Two studies with both cohort and modeling elements were evaluated with a hybrid of CHEERS and STROBE items. Overall, tools were chosen to match study design and to provide transparent, standardized assessment of bias and reporting quality.

Assignment of evaluation instruments to the studies is detailed in Supplementary Table S2 (Extraction).

Risk of bias was rated using the categories “yes”, “no”, “unclear”, and “not applicable”. These four categories were taken from the JBI tools (14, 15) and used for the ISPOR-SMDM framework (for which the respective paper does not specify categories in the form of a checklist (12)). For the QUIPS-assessment, only the categories “yes” and “no” were used, as suggested by the authors (13). This procedure was implemented to promote homogeneity and enhance comparability. Reporting quality was rated as “adequate”, “partially adequate”, “inadequate”, or “not applicable”. The exact forms used can be found in the Supplementary Tables S39–S46 (Extraction).

Items and fulfillment were depicted in tables for each study. Answers in each category were counted for each study; in this step, no overall quality score was calculated, as summarizing items into a single number can obscure important methodological strengths or weaknesses. This approach allows a transparent reflection of performance across all assessed domains. Identification and retrieval of evidence pertinent to the quality assessment in this study was facilitated by NotebookLM (Version 1.0, model notebooklm-1.0, accessed through Google's platform^2^).

To enable cross-study comparison of risk of bias and reporting quality, we quantified the proportion of assessment items in each category, indicating the distribution of ratings across studies (see Supplementary Table S4a,b (Extraction)).

Despite the employment of artificial intelligence, all final methodological decisions and quality assessments were performed by the authors. Quality assessment was conducted independently by two reviewers (XT, SJP), with disagreements resolved by consensus. Full results of the risk of bias and reporting quality assessments are provided in the Supplementary Tables S16, S18, S20, S22, S24, S26, S28, S30, S32, S34, S36, and S38 (Extraction). This review's main text will not discuss all items in detail due to the large amount of data but will provide a summary in the “Results”-section.

### 2.5 Data synthesis and comparison

Because this review focused on health states and transition probabilities, no single summary effect measure (e.g., risk ratio, hazard ratio) was predefined. Instead, transition probabilities as reported in the included studies were extracted directly. Where studies reported transition probabilities with different cycle lengths, probabilities were standardized to a common time frame, using the longest cycle length reported across studies. This approach ensured comparability across studies while minimizing the number of conversions applied and avoiding the artificial inflation of short-term fluctuations that can occur when short-cycle probabilities are extrapolated repeatedly. A standard exponential (constant-hazard) transformation was used. Specifically, if a probability *p* was reported for a cycle length of *T*, the corresponding probability $p^{\prime }$ for the target length was calculated as ${\;p}^{\prime }=1-(1-p{)}^{t/T}.$

This approach assumes that the transition hazard remains constant within and across cycles (time-homogeneity), that events occur independently within and across cycles, and that the process is memoryless, consistent with Markovian properties. These assumptions simplify the mathematical transformation and make it feasible to compare probabilities derived from studies with different cycle lengths.

Potential limitations of this method should be noted. If transition hazards vary over time, if within-cycle dynamics are complex, or if probabilities over short cycles are high (approaching 1), the conversion may over- or underestimate cumulative probabilities. Additionally, this method does not capture non-Markovian effects such as history-dependent transitions or event clustering, which may be relevant in some health states. Despite these limitations, this approach is widely used in health economic modeling and provides a transparent and reproducible method for standardizing probabilities across studies.

No pooled summary measure was calculated.

This is an adjustment to our protocol, which had specified that probabilities would be adjusted to the most commonly used cycle length. In practice, the longest cycle length was selected, as this provided a more consistent basis for comparison and reduced the number of conversions required.

To allow comparison across studies, health states were grouped according to similarity in terminology. Within each group, transition probabilities were compared when more than one study reported the same transition. If a probability was given for a control and an intervention group, it was still counted as one (kind of) transition; for synthesis, the control group was used.

- When transitions were reported in at least three studies, estimates were summarized visually in graphs to illustrate point estimates and ranges.
- When only two studies reported the same transition, absolute differences in probabilities were calculated.
- Across all 12 reports, the probability of remaining in the initial “perfect” state (e.g., healthy, natural, R0, sound) was compared, as this outcome was consistently reported.

Significant methodological and outcome heterogeneity prevented any meta-analysis or statistical pooling from being performed. A structured descriptive comparison was conducted. Subgroup comparisons correspond to the grouping of studies by terminology of heath states.

This approach expands our protocol. The protocol had specified that, where sufficient data were available, transition probabilities would be presented using forest plots. As studies lacked the report of confidence intervals or standard errors, alternative graphics were produced instead. In addition, while the protocol had envisaged using structured tables only when graphical comparison was not possible, in this review tables were used throughout: both for extraction and as a way of presenting the data that were compared across studies, sometimes in combination with graphs, and sometimes alongside narrative descriptions.

All tabulations and graphs were created in Microsoft Excel (version 2509 (Build 19231.20156 Click-to-Run)). The data can be found in the Supplementary Tables S7a,b, S9a–c, S11a,b, S13a,b, and S14 (Extraction), and the Supplementary Figures S1a−S4 (Extraction).

### 2.6 Assessment of reporting bias and certainty of evidence

Two independent reviewers (XT, SJP) assessed risk of bias due to missing results. Any disagreements were resolved by consensus. Because the number of included studies was small and the study designs and outcomes were highly heterogeneous, statistical methods for detecting reporting bias (e.g., funnel plots or regression-based asymmetry tests) were not appropriate. Instead, we adopted a two-stage qualitative approach.

We first checked whether included studies reported a study protocol or ethical approval. For those that did, we searched Supplementary Files, the Open Science Framework, and PubMed for publicly available protocols; for the single randomized controlled trial Morgan et al. (19), ClinicalTrials.gov was also checked. Protocols allow direct comparison of pre-specified vs. reported outcomes, and the search was limited to included studies following PRISMA recommendations to assess selective reporting. Availability of protocols is noted in Supplementary Table S3 (Extraction).

For studies without accessible protocols, potential selective reporting was evaluated by comparing outcomes and transitions described in methods with results, noting instances of “data not shown” or unreported outcomes. Potential conflicts of interest, such as commercial funding, were also recorded.

No formal GRADE (Grading of Recommendations Assessment, Development and Evaluation) rating was applied, as the focus was on transition probabilities rather than clinical effectiveness. Instead, we qualitatively considered certainty domains—including risk of bias, consistency, indirectness, imprecision, and potential reporting or funding bias. These assessments were not pre-specified but were added to enhance review quality and PRISMA adherence.

## 3 Results

### 3.1 Search results

**Figure 2:**
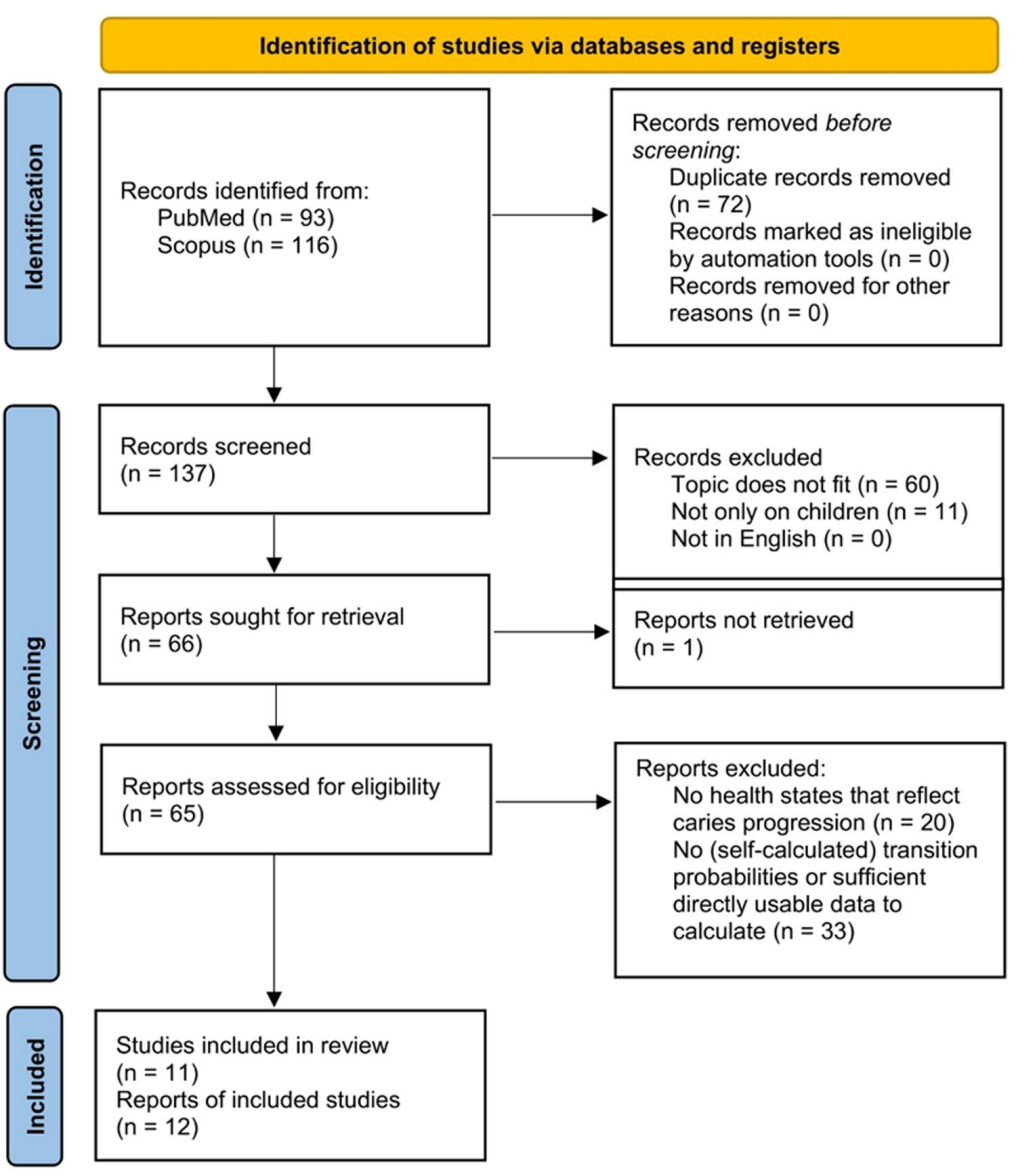
Flowchart, record inclusion.

Our search yielded 93 results on PubMed and 116 results on Scopus. Of the 116 Scopus results, 72 had already appeared in the PubMed search; after removing duplicates 137 original studies were identified for a title/abstract review. All studies were in English. We excluded 60 studies because their topic was outside the scope of this review, eleven further papers were excluded because they did not specialize in children. 66 records initially appeared to meet inclusion criteria and were selected for a full text review. One record had to be excluded because we could not retrieve the full text; 53 publications were excluded from the review because they did not provide directly usable information for the calculation of transition probabilities and/or adopted probabilities from external sources. Twelve publications on eleven studies were included in the systematic review. This is demonstrated in Figure 2.

A full table of search results and reasons for exclusion can be found in the Supplementary Material in the file “Screening”. Examples include “Conventional treatment, Hall Technique or immediate pulpotomy for carious primary molars: a cost-effectiveness analysis” by Schwendicke, Stolpe and Innes (20), which was excluded due to health states being decision-based actions, and “The natural history of dental caries lesions: A 4-year observational study” by Ferreira Zandoná et al. (21), which was excluded due to not specifying what state a surface progressed to.

### 3.2 Study characteristics and quality

The included studies varied in design, population, and duration—study characteristics can be found in the Supplementary Tables S15, S17, S19, S21, S23, S25, S27, S29, S31, S33, S35, and S37 (Extraction). Most were prospective cohort studies or clinical trials, while several employed Markov models for economic evaluations and simulation of long-term transitions. Sample sizes ranged from fewer than 200 participants in longitudinal cohorts to simulations involving millions of teeth. Follow-up periods varied from one to six years in observational studies, and up to five years in modeled analyses. The studies addressed different aspects of dental caries, including lesion progression and regression, predictors of caries activity, and the effectiveness and cost-effectiveness of preventive strategies such as fluoride varnish or pit-and-fissure sealants. A detailed overview of study objectives, designs, populations, and main findings is provided in Table 1. Notably, Dalla Nora et al. (22) and Zenkner et al. (23) draw on the same prospective cohort of 193 schoolchildren from southern Brazil and should therefore be considered two reports on a single study rather than independent sources of evidence.

**Table 1:** Characteristics of all included studies.

No.	Study ID	Title/subject of study	Objective	Study design & sample	Key characteristics/findings
1	Chi D14	Cost-Effectiveness of Pit-and-Fissure Sealants on Primary Molars in Medicaid-Enrolled Children	To compare the incremental cost-effectiveness of “always seal” and “never seal” primary molar sealant strategies with standard care for Medicaid-enrolled children.	A tooth-level Markov model was developed using Iowa Medicaid claims data (2008–2011), simulating 10,000 primary molars.	Compared to standard care, always seal was more costly but significantly reduced subsequent restorations (from 2,389 to 340). The incremental cost was $8.12 per restoration avoided.
2	Dalla21	Radiographic pattern of inactive occlusal enamel lesions (IECL) and its relationship with caries progression	To assess the radiographic pattern of inactive enamel caries lesions (IECL) on permanent molar occlusal surfaces and investigate if baseline radiolucency predicts progression over 4–5 years.	Prospective cohort study over 4–5 years. Sample included 193 schoolchildren (916 permanent molars) from southern Brazil. Logistic regression models used Generalized Estimating Equations (GEE).	The presence of radiolucency at baseline was a predictor of caries progression (adjusted odds ratio (OR) = 3.37 for activity criteria; adjusted OR = 4.01 for severity criteria).
3	Ismai11	A transition scoring system (TSS) of caries increment with adjustment of reversals in longitudinal study	To evaluate a new comprehensive Transition Scoring System (TSS) for longitudinal studies using the International Caries Detection and Assessment System (ICDAS) criteria.	Longitudinal cohort study (Detroit Dental Health Project) using primary tooth surface data. Sample consisted of 638 children, examined in 2002–03 and again in 2007.	The TSS was found to be more efficient than the modified Beck's method because it yielded a significantly smaller Coefficient of Variation (CV). TSS enabled the measurement of caries initiation and progression separately, accounting for biologically plausible reversals.
4	Kopyc06	Application of inhomogeneous Markov models for analyzing longitudinal caries risk	To apply inhomogeneous Markov models to analyze longitudinal caries risk, specifically time-dependent transition probabilities.	Longitudinal study over 6 years. Cohort of 631 caries-free children (6–7 years old) from New York. Developed two-state and three-state Markov models.	Markov modeling showed promise for analyzing caries risk. The models examined the transition from a caries-free state to a caries-active state.
5	Lawre97	Caries progression in fluoridated and fluoride-deficient areas in Brazil	To compare 1-year caries progression rates in permanent teeth in optimally fluoridated areas and fluoride-deficient areas.	Prospective epidemiologic longitudinal study over 1 year. Sample of 290 schoolchildren (12–16 years old) in Rio de Janeiro, Brazil. Caries progression evaluated using standardized bitewing radiographs and Pitts’ scoring system.	The mean rate of approximal caries progression in fluoridated areas (0.54) was 62% lower than in fluoride-deficient areas (1.41). Water fluoridation significantly contributed to slower progression rates.
6	Maltz20	Diagnosis of a Patient's Caries Activity Based on Lesion Activity Assessment	To evaluate the progression of sound surfaces, inactive non-cavitated (INC), and active non-cavitated (ANC) lesions to determine if lesion activity assessment reliably diagnoses a patient's caries activity.	Cohort study following 801 adolescents (mean age 12 at baseline) from Southern Brazil over a mean period of 2.5 years. Used Negative Binomial Regression models adjusted for clustering.	INC and ANC lesions showed similar progression risks (incidence rate ratio (IRR) 0.92). However, sound surfaces in caries-active patients had a significantly higher progression risk (IRR 2.78 vs. caries-free patients), supporting the classification of a patient's caries activity profile based on lesion features.
7	Morga08	Anticariogenic Effect of Sugar-Free Gum Containing CPP-ACP Nanocomplexes on Approximal Caries	To investigate the progression and regression of approximal caries in adolescents chewing casein phosphopeptide–amorphous calcium phosphate (CPP-ACP) gum relative to a control sugar-free gum.	Randomized, double-blind, actively controlled clinical trial (RCT) over 24 months. 2,720 adolescents (11.5–13.5 years old) from Melbourne, Australia. Used digital bitewing radiography and proportional-odds ordered logistic regression.	The CPP-ACP gum significantly slowed progression and enhanced regression of approximal caries. The odds of a surface experiencing caries progression were 18% less for the CPP-ACP group (OR = 0.82, *p* = 0.03).
8	Palac19	Assessing the cost-effectiveness of a fluoride varnish programme in Chile	To evaluate whether Fluoride Varnish application (FV) increases the proportion of caries-free children in the Chilean preschool population at an acceptable cost.	Cost-effectiveness analysis using a Markov model (Decision Analytic Model, DAM). Compared FV interventions delivered in preschool settings (PSS) or primary care settings (PCS) vs. counseling-only, over a 2-year time horizon.	FV application in a Primary Care Setting (PCS) without screening was the most cost-effective strategy. This intervention increased the prevalence of caries-free children by 3.7%.
9	Utria98	Changes in the oral health of adolescents treated by the Finnish public dental services	To monitor changes in oral health status (decayed, missing, filled (DMF), decayed (D), community periodontal index of treatment needs (CPITN) indices) in young people (13–15 years) receiving treatment through Finnish public dental services.	Follow-up study over 3 years (1992–1995). Sample consisted of 2,422 young people from four health centers in Finland.	60% of those initially caries-free did not develop cavities. Patients who initially had at least two carious teeth developed at least three new ones 50% of the time. Initial high caries status (DMF≥2 or D > 0) predicted a five times greater risk for new caries.
10	Xie Y19	Cost-effectiveness Analysis of Comprehensive Oral Health Care (COHC) for Severe Early Childhood Caries (S-ECC)	To evaluate the cost-effectiveness of comprehensive oral health care (COHC) for S-ECC in urban Beijing, China.	Randomized clinical trial (1 year duration) followed by a Markov model simulation (4 years duration, 6-month cycles). 187 children (aged 3–5 years) with S-ECC were enrolled.	COHC had extraordinary cost-effectiveness. The cumulative cost for the test group was lower than the control group when the model circulated for more than 1.5 years. After 1 year, the average fee to reduce one decayed tooth was 184 Renminbi (RMB) in the test group vs. 614 RMB in the control group.
11	Zenkn19	Long-term follow-up of inactive occlusal caries lesions: 4–5-year results	To assess the clinical behavior and estimate the risk for progression of inactive caries lesions on occlusal permanent molars compared with sound surfaces.	Prospective cohort study following 193 schoolchildren (74.8% response rate) in a low-caries prevalence population in Santa Maria, Brazil, over 4–5 years. Site-specific analyses utilizing morphological mapping were performed.	The vast majority (85%–90%) of inactive lesions did not progress. However, inactive lesions had an increased progression risk compared to sound surfaces (OR = 2.34 by activity criterion; OR = 2.69 by severity criterion).
12	Zhou23	Cost-Effectiveness of Pit and Fissure Sealing at Schools for Caries Prevention in China	To estimate the cost and effect of Pit and Fissure Sealant (PFS) application in schools in China to prevent dental caries.	Multistate Markov model analysis (5-year time frame) simulating PFS application on the Permanent First Molars (PFMs) of 13.43 million seven-year-old children, comparing it to no intervention. Analyzed from payer and societal perspectives.	PFS application was cost-effective and cost-saving from both the payer (benefit-cost ratio (BCR) = 1.22) and societal perspectives (BCR = 1.91). PFS application averted 16.06 million dental caries lesions in the PFMs.

Risk of bias and reporting quality assessment, details of which can be found in the Supplementary Materials in the file “Extraction” showed heterogeneous results. Although in all twelve reports, over half of the items were rated “yes” (risk of bias) and “adequate” (reporting quality), proportions ranged from 0.545 Utriainen et al. (24) to 0.909 Lawrence & Sheiham (25) in risk of bias assessment and from 0.5 Xie et al. (26) to 0.912 Maltz et al. (27) for reporting quality assessment. Presenting with low risk of bias did not correlate with high reporting quality and vice versa, as both highest and lowest quality publications were not the same for risk of bias and reporting quality. Still, when adding “yes” and “adequate” rates to obtain a single value that publications can be ranked by, Xie et al. (26) and Maltz et al. (27) rank lowest and highest again, respectively. Supplementary Table 4a,b (Extraction) shows rating proportions for all twelve publications and ranks them according to the sum of “yes” and “adequate” ratings.

The included studies showed several methodological limitations, though not all equally affected the assessment of health states and transition probabilities. Common issues in cohort studies included loss to follow-up and baseline differences, potentially introducing selection and attrition bias; for instance, in Maltz et al. (27) and Utriainen et al. (24), participants lost to follow-up had higher baseline caries activity, likely underestimating transition probabilities. Measurement problems such as inconsistent lesion assessment and lack of examiner calibration [Ismail, Lim & Sohn (6); Utriainen et al. (24)] also affected reliability. Some economic evaluations made simplifying assumptions—fixed transition probabilities Xie et al. (26) or independence of tooth surfaces Chi et al. (28)—which could influence estimates and uncertainty. Incomplete adjustment for confounders like diet or prior dental care Dalla Nora et al. (22); Xie et al. (26) was also noted but likely had limited impact on overall transition patterns.

Overall, the most relevant limitations for interpreting transitions between dental health states were attrition, measurement variability, and modeling assumptions, whereas other issues (e.g., missing cost components in economic analyses) have limited impact on the health-state transitions themselves.

### 3.3 Health states and transition probabilities

Building on these considerations, dental health states and transition between them will be presented in the following. A summary including health-state framework, unit of analysis, follow-up duration or cycle length, and principal transition findings can be seen in Table 2.

**Table 2:** Key model features.

No.	Study ID	Framework	Unit of analysis	Time horizon	Principal transition findings
1	Chi D14	Multi-State Models with Restored and Missing States	Tooth	Cycle: 1 month, Duration: 8.5 years	Stability dominant: 99.2% of natural teeth remained natural per cycle. Sealed teeth showed 99.7% stability with occasional sealant loss to natural (0.3%). Restored and extracted teeth were near-absorbing. Adult teeth were absorbing.
2	Dalla21	Lesion-Stage Models Based on Clinical Diagnostic Systems and Radiolucency	Tooth	4–5 years	Sound teeth without radiolucency showed only 40% stability; 51.9% transitioned to inactive non-cavitated lesions. Sound teeth with radiolucency showed even lower stability (10%), with 70% transitioning to inactive non-cavitated. Transitions predominantly occurred toward inactive rather than active states.
3	Ismai11	Lesion-Stage Models Based on ICDAS, Incorporating Reversible Lesion Dynamics	Tooth	4–5 years	Sound unerupted teeth showed 87.2% stability. Most transitions were toward early non-cavitated disease (5.4%) rather than cavitated stages (3.9%) or filled states (2.0%). Transitions to missing were rare (0.1%).
4	Kopyc06	Two-State Models of Caries Onset	Person	Cycle: 6 months, Duration: up to 6 years	Caries-free stability: 95.3–99.5% per 6-month cycle; onset of caries: 0.5%–4.7%. High MS levels elevated caries onset risk to 0.3%–9.2% vs. 0.5%–3.1% for low MS. Caries-active was absorbing. Probabilities were time-dependent.
5	Lawre97	Lesion-Stage Models Based on Radiolucency	Surface	1 year	97.1% of R0 surfaces remained stable at one year. Transitions predominantly occurred between adjacent states. Regression was observed at all radiographic stages, including 3.8% from R1 to R0 and 3.8% from R2 to R0.
6	Maltz20	Models Incorporating Reversible Lesion Dynamics	Surface	2.5 years	Sound surfaces: 93.7% stable; 4.9% transitioned to inactive non-cavitated. Inactive non-cavitated lesions showed 77.4% stability and notable regression to sound (12.9%). Active states were less stable than inactive states overall.
7	Morga08	Lesion-Stage Models Based on Clinical Diagnostic Systems	Surface	24 months	Control group: 94.7% of R0 surfaces remained stable; 2.9% progressed to R1, 1.9% to R2. CPP-ACP group showed modestly higher stability (95.7%). Regression from R1 to R0 observed in both groups. Transitions mainly occurred between adjacent states.
8	Palac19	Two-State Models of Caries Onset	Person	Cycle: 6 months, Duration: 2 years	Caries-free stability: 85.4–89.7% per 6-month cycle; caries onset: 10.3–14.6%. Caries was absorbing. Probabilities were age- and cycle-specific, derived from cross-sectional data, and notably higher than Kopyc06.
9	Utria98	Periodontal Health State Model	Person	2–3 years	All three periodontal states showed bidirectional transitions with no absorbing states. Healthy: 47% stable, 24.6% progressed to affected. Affected: 48.3% stable, 18.9% regressed to healthy. Substantial movement in both directions across all states.
10	Xie Y19	Multi-State Models with Restored and Missing States	Person (for the intervention), tooth (for the model)	Cycle: 6 months, Duration: 4 years	Natural teeth: 98.2% stable (control). Decayed teeth: 90.7% stable, with 9.1% transitioning to restored in controls vs. 31.3% in the intervention group. Restored teeth were not fully absorbing (4.7% reverted to decayed). Missing was absorbing.
11	Zenkn19	Models Incorporating Reversible Lesion Dynamics	Surface	4–5 years	Sound surfaces: 38.3% stable over 4–5 years; 53.1% transitioned to inactive non-cavitated. Inactive non-cavitated: 63.8% stable, with 9.6% regression to sound. Active states showed greater progression toward cavitation than inactive states.
12	Zhou23	Multi-State Models with Restored and Missing States	Tooth	Cycle: 1 year, Duration: 5 years	Natural teeth: 70.8% stable in controls (lowest among multi-state models); 29.2% progressed to decayed. PFS intervention raised stability to 83.8%–98.8%. Decayed: 83.6% stable; restored: 89.3% stable with 10.7% reverting to decayed. Missing was absorbing.

The included twelve reports on eleven studies (cohort, modeling, and one RCT) examined transitions between dental health states with heterogeneous model structures, diagnostic classifications, and follow-up periods. Despite these methodological differences, several common patterns emerged regarding the definition of health states and the dynamics between them.

#### 3.3.1 Multi-state models with restored and missing states

Several studies used multi-state models describing transitions between mutually exclusive dental states. For example, Chi, van der Goes & Ney 2014 (28) modeled five mutually exclusive health states (“natural”, “sealed”, “restored”, “extracted”, and “adult” teeth), with “adult” and “extracted” as absorbing states (states from which no further transitions are possible, such as tooth extraction). Across these models, remaining in the same state was the most common outcome, indicating that transitions representing disease progression or treatment occurred relatively infrequently within the observed time frames.

Similar state structures were applied by Xie et al. 2019 (26) and Zhou et al. 2023 (29), which modeled transitions among “natural”, “decayed”, “restored”, and “missing” states, each including an absorbing outcome. Consistent with other multi-state models, the probability of remaining in the same state was highest, while progression transitions occurred less frequently. However, notable differences between studies were observed for transitions such as “natural → decayed” and “decayed → restored”, reflecting variations in study populations, follow-up duration, and treatment patterns (see Supplementary Figure S2 (Extraction)).

#### 3.3.2 Lesion-stage models based on clinical diagnostic systems

Other studies applied more detailed lesion-stage classifications, particularly those based on clinical diagnostic systems such as ICDAS or lesion activity status. For instance, Ismail et al. (6) applied ICDAS-based states over four to five years, showing high persistence within states (>80%) and limited reversibility between active and inactive lesions. Similarly, Dalla Nora et al. (22) distinguished cavitated and non-cavitated lesions, active/inactive stages, and radiolucency. In these models, transitions occurred predominantly among early, inactive stages, while cavitated states were largely stable (6). A number of studies employed radiographic scoring systems to define health states. Lawrence & Sheiham (25) and Morgan et al. (19) used radiographic R-score classifications (R0-R4 and R0-R8, respectively). Both studies found that transitions most frequently occurred between adjacent radiographic states, while stability of the initial state remained the dominant outcome. Occasional regression between states was also observed.

#### 3.3.3 Two-state models of caries onset

Other models simplified disease representation using two-state frameworks, typically distinguishing between sound and diseased teeth. For example, Palacio et al. (30) modeled transitions between caries-free and caries-active states. Similarly, Kopycka-Kȩdzierawski & Billings (31) contributed two distinct models: the first used a basic two-state framework (sound vs. diseased), while the second separated caries-free teeth into high and low levels of mutans streptococci to capture additional heterogeneity. Although both assumed irreversible caries onset, the reported transition probabilities differed substantially, likely reflecting differences in study populations, caries risk levels, and follow-up durations (see Supplementary Figures 1a,b (Extraction)).

#### 3.3.4 Models incorporating reversible lesion dynamics

Several studies also explicitly incorporated reversible lesion dynamics, allowing transitions between active and inactive states as well as restored outcomes. For example, Maltz et al. (27), Zenkner et al. (23), and Ismail et al. (6) modeled reversible transitions reflecting the dynamic nature of caries activity. Differences between studies primarily concerned the frequency of lesion reactivation or progression.

#### 3.3.5 Periodontal health state model

One study diverged from tooth-based or lesion-based frameworks by modeling periodontal health states at the sextant level. Utriainen et al. (24) reported transitions between “healthy”, “slightly affected”, and “affected” gingival states, with moderate movement between all categories over time. Although conceptually related to oral health progression, this model differs substantially from caries-focused frameworks and therefore contributes limited direct comparability to the transition structures used in the other included studies.

#### 3.3.6 Overall synthesis

Despite these common patterns, substantial heterogeneity was observed in the definition of health states, diagnostic classifications, cycle lengths, and modeling assumptions. These methodological differences limit the comparability of reported transition probabilities across studies and complicate the direct synthesis of results. The variability in state definitions and reporting practices also highlights the lack of standardized approaches for modeling dental caries progression in pediatric populations.

The variability in transition probabilities across models is visualized in Supplementary Figures 3a–b (Extraction), while the probability of remaining in an initial “perfect” state (e.g., sound, natural, or R0) across studies is shown in Supplementary Figure S4 (Extraction). Detailed comparison data are provided in Supplementary Tables S5–S14 (Extraction).

Due to the considerable methodological and clinical heterogeneity between studies, a meta-analysis or quantitative pooling of transition probabilities was not performed. Instead, the synthesis was conducted descriptively, comparing transition probabilities and contextual study characteristics. Formal heterogeneity testing was not applicable as no pooled estimate was calculated. Sensitivity analyses were not performed given the descriptive nature of the synthesis.

### 3.4 Reporting bias and certainty of evidence

Potential reporting bias was explored. Eight of twelve publications mentioned a protocol or predefined outcomes, but none provided accessible registrations. Two studies Chi et al. (28); Lawrence & Sheiham (25) omitted predefined outcomes (“avoided extractions” and “net whole-mouth score”). Two Morgan et al. (19); Maltz et al. (27) reported commercial funding. These issues indicate possible selective-reporting risk.

Overall, certainty of evidence was moderate. Empirical studies showed selection and attrition bias, as participants lost to follow-up often had higher baseline caries activity. Measurement and examiner-calibration issues further reduced reliability. Modeling studies were limited by parameter uncertainty, simplifying assumptions (e.g., fixed transition probabilities, independence of teeth), and limited data sources.

The data suggests an observed stability in dental health states, though this pattern may be influenced by the loss of high-risk participants (attrition bias) noted in the primary studies. Nevertheless, comparability across studies is limited. Methodological weaknesses, reporting gaps, and model assumptions warrant cautious interpretation.

## 4 Discussion

### 4.1 Findings on health states and transition probabilities

This systematic review synthesized transition probabilities between dental health states in children across 12 publications encompassing observational cohorts, one randomized controlled trial, and modeling analyses. Overall, stability within the same health state was the predominant pattern, while transitions representing disease progression or regression occurred less frequently. Regression (i.e., as the transition from active to inactive lesions) were primarily observed in models explicitly accounting for lesion activity.

Simple two-state models, including Palacio et al. (30) and Kopycka-Kȩdzierawski & Billings (31), captured caries initiation as a largely unidirectional process, whereas more complex multi-state models, such as Ismail et al. (6), Maltz et al. (27), and Zenkner et al. (23), allowed for bidirectional transitions and differentiated between lesion depth and activity. The probability of remaining in a “perfect” or “healthy” state was generally high (>99%) in most publications, although some reported lower probabilities (∼40%–87%), reflecting population differences, follow-up duration, and model assumptions.

The exceptionally high stability observed in pediatric dental caries highlights the slow, modifiable nature of disease progression and the potential for preventive interventions to sustain oral health over extended periods. This principle—that targeting high-risk subgroups is often more efficient—aligns with broader findings from chronic disease modeling, emphasizing that early identification of high-risk individuals and monitoring of modifiable factors improves intervention efficiency. In pediatric dental caries, this supports the focus on children with elevated baseline risk or early-stage lesions when designing preventive programs.

Differences in model structure, follow-up periods, population characteristics, and definitions of health states resulted in substantial heterogeneity across studies, limiting comparability. Despite this, consistent patterns emerged: early-stage lesions were more likely to progress to inactive or minor cavitated states than to advanced cavitation or extraction, and restored or missing states generally acted as absorbing states. Remaining in the same health state appears to trend toward the most common outcome among the included studies, though this observed stability should be interpreted alongside the reported attrition bias of high-risk individuals. Progression, regression, and reversals occurred less often. Variability in cycle lengths and follow-up periods further limited direct comparability of transition probabilities. These findings suggest that pediatric dental caries progression is predominantly stable over short- to medium-term follow-up, with meaningful but limited transitions to more severe states.

Comparing studies highlights the influence of methodological choices on observed transition probabilities. Simple two-state models primarily depict disease onset, with a single irreversible transition from “caries-free” to “caries-active” states. Palacio et al. (30), who calculated age-specific transition probabilities, reported higher disease onset probabilities than Kopycka-Kȩdzierawski & Billings (31), likely reflecting differences in follow-up interval, population risk profile, and underlying assumptions.

In contrast, multi-state models provided a more nuanced picture of lesion progression and regression, capturing both active and inactive lesions and allowing for restorative interventions. Maltz et al. (27) and Zenkner et al. (23) reported bidirectional transitions between sound, inactive, and active non-cavitated lesions, demonstrating the potential for remineralization and highlighting the value of detailed lesion-level modeling. Only two out of 13 proposed models^3^ Kopycka-Kȩdzierawski & Billings (31), Palacio et al. (30) consisted solely of caries-free and caries-active states, reflecting a trend toward the use of multi-state models among the 12 included publications, which allow for more granular transition mapping than simpler frameworks within this limited sample. Evidence from other chronic disease modeling suggests that multi-state frameworks are generally preferred when transitions are heterogeneous, supporting their methodological suitability for pediatric dental caries.

Transition patterns were also influenced by population characteristics and baseline risk. In studies with higher baseline caries activity or high-risk populations, such as Kopycka-Kȩdzierawski & Billings (31), transition probabilities from caries-free to active caries states were increased. Conversely, interventions like pit-and-fissure sealants Zhou et al. (29) significantly reduced the likelihood of progression from sound to decayed states, demonstrating that preventive strategies can meaningfully alter transition dynamics.

Remaining in the same health state was consistently the most common outcome across models, highlighting the overall stability of dental health in the populations studied. Progression to cavitated, restored, or missing states was less frequent, but the specific probability varied across studies due to differences in definitions, measurement methods, and cycle lengths. For example, Xie et al. (26) and Zhou et al. (29) shared similar transitions but differed substantially in probabilities for “natural” to “decayed” and “decayed” to restored transitions, with differences up to 26 and 16 percentage points, respectively. These variations underscore the importance of standardizing health-state definitions and measurement approaches to improve comparability and model accuracy.

Variability in cycle lengths and follow-up periods across studies further limited direct comparability of transition probabilities.

### 4.2 Limitations of studies

Several methodological limitations were observed across studies, influencing both the reliability of reported transition probabilities and the certainty of the evidence. In cohort studies, loss to follow-up and baseline differences were common sources of potential bias. Attrition bias is a common issue in longitudinal studies, arising when participants drop out in a non-random manner. This can distort the sample and lead to underestimation of transition probabilities, particularly if dropout is related to health status or other key outcomes (32). Participants lost to follow-up often had higher baseline caries activity, as observed in Maltz et al. (27) and Utriainen et al. (24). Selection bias was compounded in some studies by differences in baseline characteristics between groups, limiting internal validity and comparability.

Measurement variability also represented a key limitation. Inconsistent lesion assessment methods, lack of examiner calibration, and differing criteria for activity and cavitation (e.g., ICDAS vs. radiographic scoring) introduced variability in observed transitions. Utriainen et al. (24) highlighted that diagnoses resulting from routine examinations as part of a dentist's everyday work may differ between examiners. Ismail et al. (6) and Utriainen et al. (24) demonstrated how differences in classification systems could lead to divergent estimates of lesion progression and regression, emphasizing the impact of measurement heterogeneity on transition probabilities.

In modeling studies, structural assumptions affected the robustness of estimated probabilities. Several models assumed fixed transition probabilities, independence of tooth surfaces, or complete adherence to preventive interventions, potentially under- or overestimating transitions. Fixed transition probabilities can be particularly limiting because they assume that the likelihood of moving between states remains constant over time, which may not reflect the true dynamics of disease progression and can lead to inaccurate predictions (33). For example, Xie Y19 assumed fixed probabilities for all surfaces, while Chi et al. (28) treated the “adult tooth” as an absorbing state disconnected from other transitions. Dalla Nora et al. (22) accounted for active/inactive lesion states with and without radiolucency, highlighting the potential for more accurate estimates but also demonstrating the complexity introduced by detailed state definitions.

Incomplete adjustment for confounders, including dietary habits and prior dental care utilization, further reduced certainty. Dalla Nora et al. (22) and Xie et al. (26) were limited in controlling for these factors, which may influence lesion progression and stability. Reporting limitations were also evident: no study had a publicly accessible protocol, and several omitted pre-specified outcomes, introducing potential selective reporting bias. Two studies Morgan et al. (19) and Maltz et al. (27) reported commercial funding, which may further contribute to selective reporting.

Across the included studies, confidence intervals (CIs) were generally not reported for the raw transition probabilities or observed transition frequencies. Instead, CIs were typically provided only for derived outcomes, such as incremental cost-effectiveness ratios, odds ratios, or regression coefficients, to quantify uncertainty in these estimates. For studies using Markov models [e.g., Chi et al. (28); Kopycka-Kȩdzierawski & Billings (31); Xie et al. (26); Palacio et al. (30)], transition probabilities were used as point estimates or modeled with probability distributions (e.g., Beta distributions) for probabilistic sensitivity analyses, but the input probabilities themselves were not accompanied by CIs. Similarly, in cohort studies analyzing relative risks of progression [e.g., Dalla Nora et al. (22)/Zenkner et al. (23); Morgan et al. (19); Ismail et al. (6)], odds ratios were presented with 95% CIs, whereas the underlying transition frequencies remained unaccompanied by interval estimates. This absence of CIs for the transition probabilities themselves limits the ability to quantify statistical uncertainty directly at the level of health-state changes. Reporting CIs directly for transition probabilities should be a priority in future studies to improve transparency and support probabilistic modeling.

Despite the widespread use of Markov models in health economic evaluations, there remains a notable lack of formal consensus on the estimation of transition probabilities (TPs). A review by Olariu et al. (34) highlighted that many studies fail to report uncertainty intervals, such as confidence intervals (CIs), for these estimates, which limits transparency and the robustness of decision-making (34). This issue is echoed in more recent work by Srivastava et al. (35), who reviewed 28 NICE Technology Appraisals and found that TP estimation often suffers from methodological inconsistencies, including missing transitions, reliance on heterogeneous data sources, and extrapolation beyond observed data. Importantly, uncertainty associated with TP estimation methods was rarely addressed, and sensitivity analyses typically did not account for this source of error. These findings underscore the need for clearer methodological guidance and standardized reporting practices in health economic modeling (35). Additionally, Gidwani et al. (36) provide practical guidance for estimating transition probabilities from published evidence, noting that such data are frequently presented in formats (e.g., relative risks, odds ratios) that require transformation. Crucially, they highlight that these estimates are commonly reported without accompanying confidence intervals (CIs), which limits the ability to assess uncertainty and undermines model robustness (36).

### 4.3 Existing literature

Previous reviews have generally reported similar findings regarding the predominance of stable dental health states and the relatively low frequency of lesion progression or regression in pediatric populations. Our review extends this literature by providing a structured comparison of transition probabilities across a diverse set of study designs and health-state definitions. By explicitly comparing simple and complex models, we highlight how methodological choices influence observed transitions and the representation of reversible processes.

### 4.4 Inferences for future research

The evidence synthesized in this review informs both clinical practice and health economic modeling. High stability of the “perfect” state suggests interventions targeting high-risk subgroups may be most efficient, as most children maintain healthy teeth without intensive treatment. Stability also supports the use of longer cycle lengths in Markov models without substantially underestimating disease progression, simplifying modeling while preserving accuracy.

Multi-state models, particularly those distinguishing lesion activity, appear to provide a closer approximation of real-world dynamics than simple two-state models. The observation that active lesions can regress to inactive states aligns with longitudinal cohort data and supports the biological plausibility of remineralization processes. Conversely, simple unidirectional models may overestimate disease progression and limit the ability to evaluate preventive interventions.

Understanding transition probabilities aids in designing risk-based recall intervals and targeted preventive strategies and informs policymakers evaluating interventions like fluoride varnish or sealants.

Looking forward, several priorities emerge for future research to strengthen the evidence base on pediatric dental caries transition probabilities. Standardization of health-state definitions and scoring criteria is critical to improve comparability across studies. Longer follow-up studies, particularly in high-risk populations, are needed to capture less frequent transitions and regressions. Transparent reporting, including publicly accessible protocols and full presentation of pre-specified outcomes, will reduce potential bias and facilitate meta-analytic synthesis. Future studies should also report confidence intervals or other measures of uncertainty directly for transition probabilities, rather than only for derived outcomes, to improve transparency and support probabilistic modeling. Finally, data sharing for modeling studies would also enhance reproducibility and allow validation of estimated transition probabilities across populations and interventions.

### 4.5 Strengths and limitations

This review has several notable strengths and limitations that should be considered when interpreting the findings. Strengths include the comprehensive search strategy, inclusion of both observational and modeling studies, and structured descriptive comparison of transition probabilities across multiple health-state definitions. Limitations include the inability to conduct meta-analysis due to substantial methodological and clinical heterogeneity, variability in cycle lengths and follow-up durations across studies, and potential publication bias. Additionally, standardizing transition probabilities to a common cycle lengths required assumptions of constant hazards and independent transitions, which may not fully reflect real-world dynamics and could lead to slight over- or underestimation of cumulative probabilities. Although non-English studies were not excluded, reliance on published results and Supplementary Materials may have introduced bias, as not all results may have been fully accessible. This is consistent with evidence of selective publication and reporting of favorable results in the scientific literature (37). Despite these limitations, the review provides a detailed overview of dental health state dynamics in children and identifies areas for methodological improvement.

### 4.6 Implications for clinical practice and policy

The evidence synthesized in this review has important implications for clinical practice and policy. High stability of the “perfect” or healthy state suggests that interventions targeting high-risk subgroups may be most efficient, as most children maintain healthy teeth without intensive treatment. Multi-state models that capture reversible lesion activity appear to provide a closer approximation of real-world dynamics and can inform health economic evaluations, resource allocation, and the design of targeted preventive strategies such as risk-based recall intervals and intervention planning. Understanding transition probabilities also allows policymakers to estimate the potential benefits of preventive interventions, including fluoride varnish or sealants, more accurately.

## 5 Conclusion

This systematic review provides a comprehensive synthesis of transition probabilities between childhood dental health states across 12 publications, encompassing observational cohorts, a randomized controlled trial, and modeling analyses. The results from the reviewed studies indicate an observed stability within the same health state, a pattern potentially inflated by the attrition bias (loss of high-risk participants) documented in the source literature. Particularly for the “perfect” or “sound” state, where stability often exceeds 99% over a 4–5-year period. While progression and regression occur less frequently, their representation is largely dependent on model complexity. Multi-state models that distinguish between lesion depth and activity appear to capture bidirectional transition, including regression and remineralization, whereas simpler two-state models tend to reflect primarily unidirectional disease onset and may overestimate progression.

However, the robustness of these findings is tempered by significant methodological and reporting limitations. Attrition and selection bias in cohort studies—where high-risk participants were more likely to drop out—may lead to an underestimation of transition probabilities. Furthermore, measurement variability arising from inconsistent lesion assessment and a lack of examiner calibration across studies limits direct comparability. In modeling studies, the reliance on simplifying assumptions, such as fixed transition probabilities and the independence of tooth surfaces, alongside a widespread failure to report confidence intervals for transition parameters, further reduces the certainty of the evidence.

Despite these challenges, the review offers inferences that appear to support future research, clinical practice, and policy. The high stability of early dental health suggests that preventive interventions should be strategically targeted at high-risk subgroups to maximize efficiency. For researchers, the findings suggest that multi-state models provide a more biologically plausible approximation of caries dynamics than two-state frameworks. Moving forward, the field requires harmonized health-state definitions, standardized assessment procedures, and transparent reporting—including the routine inclusion of uncertainty measures like confidence intervals—to strengthen the reliability of model-based findings and better inform evidence-based public health strategies.

## Data Availability

The original contributions presented in the study are included in the article/Supplementary Material, further inquiries can be directed to the corresponding author/s.

## Footnotes

^1^ https://chat.openai.com/

^2^ https://notebooklm.google.com

^3^There were 12 publications, but Kopycka-Kȩdzierawski & Billings (19) compared two models – (a) consisted of two health states, (b) separated caries-free states into high and low levels of mutans streptococci.
